# Chiral ruthenium(ii) complex as potent radiosensitizer of ^125^I through DNA-damage-mediated apoptosis†

**DOI:** 10.1039/c8ra03383h

**Published:** 2018-06-06

**Authors:** Mingjun Bai, Zhaolin Zeng, Li Li, Qiong Wu, Yanyang Zhang, Tao Pan, Luwen Mu, Duo Zhu, Shouhai Guan, Qiang Xie, Wenjie Mei

**Affiliations:** Department of Vascular Interventional Radiology, The Third Affiliated Hospital, Sun Yat-sen University 600 Tianhe Road Guangzhou Guangdong China 510630 xieqiangdoctorxie@126.com Guanshouhai1148598268@qq.com; School of Pharmacy, Guangdong Pharmaceutical University Guangzhou China 510006 wuqiongniu.1113@163.com

## Abstract

A chiral ruthenium(ii) complex, Λ-[Ru(bpy)_2_(*o*-tFMPIP)] (ClO_4_)_2_ (*o*-tFMPIP = 2′-trifluoromethylphenyl) imidazo [4,5-*f*][1,10]phenanthroline, was prepared and evaluated for its enhancement of the radiosensitivity of ^125^I seeds. The synthetic Ru(ii) complex, LR042, effectively enhanced growth inhibition against HepG2 human hepatocellular liver carcinoma cells induced by ^125^I seeds and consequently effectively promoted the apoptosis of tumor cells with increasing level of cleave-caspase-3. Furthermore, the results of immunofluorescence indicated that LR042 enhanced the phosphorylation of H2AX by ^125^I seeds vigorously in response to damaged DNA. LR042 improved DNA damage induced by ^125^I seeds, which resulted in apoptosis through the activation of the p53/AKT signal. In conclusion, synthetic LR042 can be further developed as a potential radiosensitizer of ^125^I seed radiotherapy for cancer therapy.

## Abstract 2

Synthetic LR042 can be further developed as a radiosensitizer of ^125^I by inducing DNA-damage-mediated apoptosis for cancer therapy.

## Graphical abstract

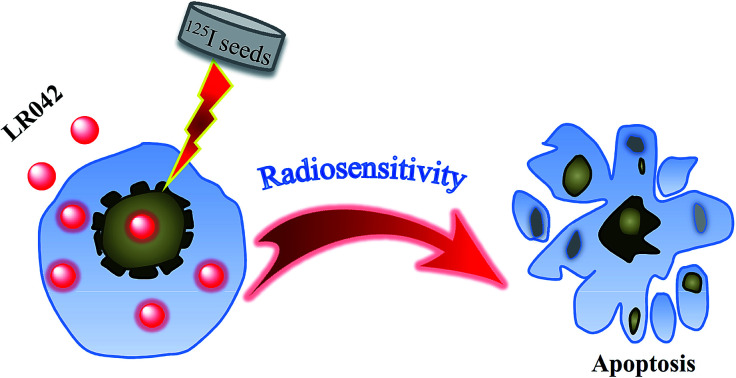

## 1. Introduction

For decades, radiotherapy is one of the most common and effective tumor therapy techniques in clinical use. Generally, radiotherapy is commonly used as a clinical noninvasive means to treat tumors. High-energy X-rays or γ-rays kill tumor cells by inducing DNA damage or causing radical damage that in turn breaks down the DNA.^1,2^ However, the application of radiotherapy technology to cancer treatment has many limitations. Radiotherapy has inefficient ability against cancer cells, because of the strong resistance of cancer cells to external beam radiotherapy (EBRT),^3^ and high rate of local recurrence.^4^ Furthermore, adjacent normal tissues could be damaged in the process of radiation treatment.^5^ Therefore, exploring a new treatment modality for tumor patients is necessary to overcome the effect of EBRT and reduce its side effects on the surrounding normal tissue.

^125^I seeds have an average energy of 27.4–31.4 keV, and their valid radius is 1.7 cm in tissue.^6^ They have been widely applied for permanent implantation in the treatment of cancers due to their high precision and low complication rate.^7^^125^I seeds can reduce the damage to the surrounding normal tissues and medical personnel. When low-energy ^125^I seeds are implanted, the gamma rays can be focused on the target area.^8^ Thus, ^125^I seeds have been applied to the treatment of head and neck carcinoma, recurrent colorectal cancer.^9,10^ However, ^125^I seed radiotherapy still cannot eradicate hypoxic tumors efficiently due to their insensitivity to radiation.^11^

To enhance the sensitivity to ^125^I seed radiation, the combination of chemotherapy and radiotherapy has become a standard treatment option.^12^ It's reported that gold nanoparticles (GNPs) can enhance radiotherapeutic efficacy of ^125^I and use as tumor-targeted radiosensitizer in oncotherap.^13^ Currently, ruthenium(ii) complexes, which show excellent inhibitory activity against various tumors but low cytotoxicity toward human normal cells, have been widely studied for their potential utility in chemotherapy. Increasing numbers of ruthenium(ii) complexes have been developed as potential anticancer drugs. For instance, NAMI-A and KP1019 have been successfully entered into clinical trials.^14,15^ Gasser and coworkers found that polypyridyl ruthenium(ii) complexes can develop into potential apoptosis inducers of cancer cells.^16^ Different ruthenium complexes have anticancer effects against a variety of cancer cells, especially against metastatic cancers.^17–19^ Our previous study found [Ru(phen)_2_(*p*-tFMPIP)](ClO_4_)_2_ as a potential dual functional agent for the inhibition of the proliferation of tumor cells through stabilizing *c-myc* G4 DNA.^20^ Therefore, ruthenium(ii) complexes may also be candidate agents for radiosensitization.

**Scheme 1:**
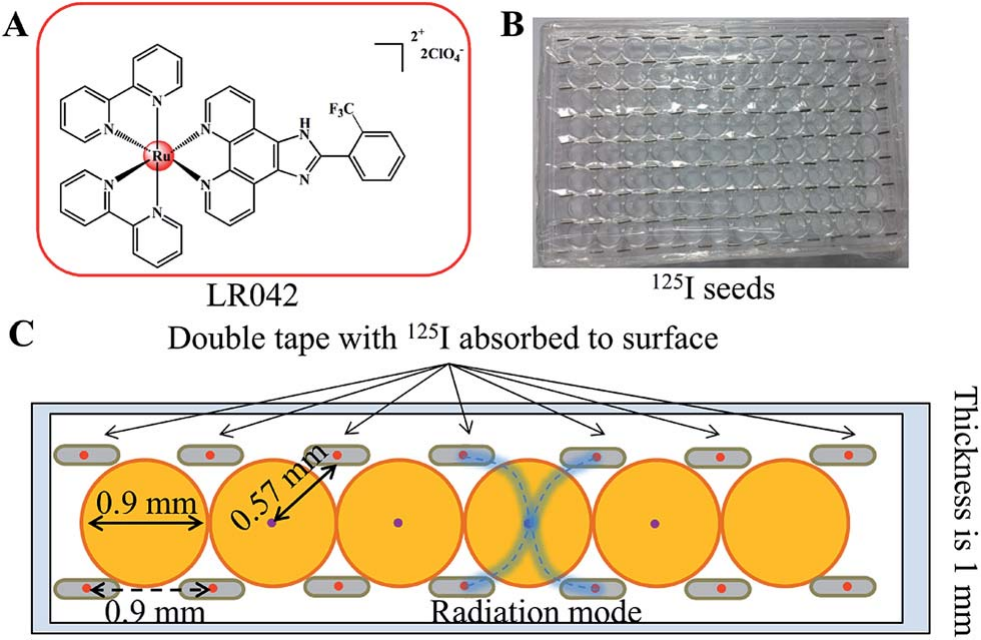
(A) Chemical structure of the target ruthenium(ii) polypyridyl complex of LR042. (B) Application diagram of the model of the ^125^I seed brachytherapy source. (C) Schematic diagram of the experimental setup for the measurement of radial dose function for ^125^I seeds. The ^125^I seeds were arranged regularly on the top of the 96 well plate.

Inspired by these findings, the present study synthesized a chiral ruthenium(ii) complex and found that such a complex can effectively enhance the ^125^I-induced growth inhibition against human hepatocellular liver carcinoma (HepG2) cells through the induction of apoptosis by triggering DNA damage. The complex also activated p53 signaling pathways to enhance the anticancer efficacy of radiation. In summary (Scheme 1), synthetic LR042 is a promising radiation sensitizer for ^125^I seed radiotherapy.

## 2. Experimental

### 2.1 Materials and methods

All reagents and solvents were purchased commercially and used without purification unless noted. Distilled water was used in all experiments. All aqueous solutions were prepared with double-distilled water. Ruthenium(iii) chloride hydrate was purchased from Mitsuwa Chemical. *cis*-Ru(bpy)_2_Cl_2_·2H2O, [Ru(bpy)_2_(py)_2_]Cl_2_, and [Ru(bpy)_2_(py)_2_][*O*,*O*′-dibenzoyl-l-tartrate]·12H_2_O were prepared and characterized according to the literature.^21,22^ Microwave-assisted synthesis was performed using an Anton Paar Monowave 300 microwave reactor. ESI-MS spectra were obtained in methanol on an Agilent 1100 ESI-MS system operated at room temperature. Cellular localization and immunofluorescence experiments were performed with an LSM800 (Germany).

### 2.2 Synthesis of (2′-trifluoromethylphenyl) imidazo [4,5-*f*][1,10]phenanthroline. (*o*-tFMPIP)

*o*-tFMPIP was synthesized according to the literature procedures^23^ with some modification. A mixture of 1,10-phenanthroline-5,6-dione (315.06 mg, 1.50 mmol), 2′-trifluoromethylphenylaldehyde (339.81 mg, 2.25 mmol), ammonium acetate (4 g, 51.9 mmol), and glacial acetic acid (20 mL) was heated at 100 °C for 20 min under microwave radiation. pH value was adjusted to 7.0 at room temperature. The solution was filtered and dried in vacuum to obtain a yellow precipitate, which was collected and washed with water and small amounts of ethanol. The crude product dissolved in ethanol was purified by filtration on a silica gel column (60–100 mesh). ESI-MS (in DMSO, *m*/*z*): 365.7 ([M + H^+^]^+^, calc. 365.1). ^1^H NMR (in DMSO-d_6_, *δ*/ppm): 9.03 (2H, d); 8.88 (2H, d); 7.99 (2H, d); 7.87 (4H, m).

### 2.3 Synthesis of Λ-[Ru(bpy)_2_(*o*-tFMPIP)] (ClO_4_)_2_. (LR042)

Λ-[Ru(bpy)_2_(*o*-tFMPIP)] (ClO_4_)_2_ (LR042) was synthesized following the literature procedure with some modifications.^24^ A mixture of [Ru(bpy)_2_(py)_2_][*O*,*O*′-dibenzoyl-l-tartrate]·12H_2_O (520 mg, 0.4 mmol), *o*-tFMPIP (188.4 mg, 0.6 mmol), and ethylene glycol (54 mL) was irradiated with microwaves for 25 min at 130 °C. The cooled reaction mixture was diluted with water. The sodium perchlorate added to the filtered liquor produced an orange suspended solid. The dark-red solid was collected, washed with water and small amounts of ethanol, dried under vacuum, and purified using column chromatography on alumina. The solvent was removed under reduced pressure, and red microcrystals were obtained.^25^ ESI-MS (in CH_3_CN, *m*/*z*): 389.3 ([M–2ClO_4_^−^]^2+^, calc. 389.1). ^1^H NMR (in DMSO-d_6_, *δ*/ppm): 9.01 (d, *J* = 8.2 Hz, 2H), 8.87 (dd, *J* = 22.5, 8.2 Hz, 4H), 8.23 (td, *J* = 8.1, 1.4 Hz, 2H), 8.12 (td, *J* = 8.1, 1.4 Hz, 2H), 8.10–8.04 (m, 4H), 7.94 (dt, *J* = 8.2, 6.0 Hz, 4H), 7.87 (dd, *J* = 10.8, 5.6 Hz, 4H), 7.67–7.64 (m, 4H), 7.63–7.57 (m, 4H), 7.38–7.34 (m, 4H). ^13^C NMR (151 MHz, DMSO) *δ* 155.14 (s), 154.89 (s), 149.93 (s), 149.76 (s), 148.20 (s), 143.45 (s), 136.23 (s), 136.09 (s), 131.03 (s), 130.80 (s), 128.69 (s), 126.24 (s), 126.13 (s), 124.77 (s), 122.76 (s).

### 2.4 Synthesis of Δ-[Ru(bpy)_2_(*o*-tFMPIP)] (ClO_4_)_2_ (DR042)

Δ-[Ru(bpy)_2_(*o*-tFMPIP)] (ClO_4_)_2_ (DR042) was synthesized by above method, but [Ru(bpy)_2_(py)_2_][*O*,*O*′-dibenzoyl-l-tartrate]·12H_2_O (520 mg, 0.4 mmol) was replaced by [Ru(bpy)_2_(py)_2_][*O*,*O*′-dibenzoyl-d-tartrate]·12H_2_O (520 mg, 0.4 mmol). ESI-MS (in CH_3_CN, *m*/*z*): 876.6 ([M–ClO_4_^−^]^+^, calc. 877.1), 776.9 ([M–2ClO_4_^−^–H^+^]^+^, calc. 777.1), 388.5 ([M–2ClO_4_^−^] ^2+^, calc. 389.1). ^1^H NMR (500 MHz, DMSO) *δ* 9.01 (d, *J* = 8.2 Hz, 1H), 8.88 (dd, *J* = 18.7, 8.2 Hz, 2H), 8.23 (td, *J* = 8.0, 1.4 Hz, 1H), 8.13 (td, *J* = 8.0, 1.4 Hz, 1H), 8.10–8.05 (m, 2H), 7.97–7.91 (m, 2H), 7.88 (dd, *J* = 10.7, 6.6 Hz, 2H), 7.66 (d, *J* = 5.1 Hz, 1H), 7.63–7.58 (m, 1H), 7.36 (ddd, *J* = 7.2, 5.8, 1.1 Hz, 1H). ^13^C NMR (151 MHz, DMSO) *δ* 155.16 (s), 154.91 (s), 149.99 (s), 149.73 (s), 148.10 (s), 143.58 (s), 136.36 (s), 136.10 (s), 131.06 (s), 130.87 (s), 128.63 (s), 126.25 (s), 126.16 (s), 124.91 (s), 122.88 (s).

### 2.5 Cell culture

The cell lines used in this study, including the human hepatocellular liver carcinoma (HepG2) cell line, human colon cancer (SW480) cell line, and human lung adenocarcinoma (A549) cell line, were purchased from the American Type Culture Collection (ATCC, Manassas, VA). All cell lines were maintained in a Roswell Park Memorial Institute medium: Dulbecco's modified Eagle medium supplemented with bovine serum albumin (BSA; 10%), penicillin (100 units per mL), and streptomycin (50 units per mL) at 37 °C in a CO_2_ incubator (95% relative humidity and 5% CO_2_).

### 2.6 MTT assay

Cell viability was determined by measuring the ability of cells to transform MTT to a purple formazan dye.^26^ Cells were seeded in 96-well tissue culture plates for 24 h. The antiproliferative effects of ruthenium(ii) complex and I^125^ were assessed using MTT assay. The cells were incubated with different concentrations of ruthenium(ii) complex (0–20 μM) with and without the treatment of I^125^ seed radiation. The ^125^I seeds were arranged regularly on the top of the 96 well plate, which keep a certain distance (0.9 cm) between two seeds. Then, the 96 well plate containing cancer cells covered on the ^125^I seeds, which cancer cells were radiated in a distance about 1 mm (the thickness of 96 well plate) with ^125^I seeds, and every well was located in the center of four seeds, which keep the well with same radiation energy. Thereafter, the cells were cultured for 72 h. At the end of incubation, 20 μL per well of MTT solution (5 mg mL^−1^ in phosphate-buffered saline (PBS)) was added and incubated for 5 h.^27^ The medium was aspirated and replaced with 150 μL per well of DMSO to dissolve the formazan salt formed. The color intensity was measured at 570 nm using a microplate spectrophotometer (Versa Max). The cell viability of the treatment groups was expressed as the percentage of the control.

### 2.7 Cellular localization

HepG2 cells in a complete growth medium at 5 × 10^4^ cells per mL were incubated with LR042 (5 μM) for 24 h at 37 °C unless otherwise stated. Cells were washed three times with PBS, fixed, permeabilized simultaneously by using 4% *p*-formaldehyde, and stained with DAPI (0.5 μg mL^−1^) for 15 min. The cell morphology was observed using a confocal laser microscope.

### 2.8 Flow cytometric analysis

The cell cycle distribution and the apoptosis rate were analyzed using flow cytometry as previously described.^28^ After incubating with different concentrations of LR042 (0, 5, and 10 μM), LR042 (0, 5, and 10 μM), and ^125^I for 72 h, the cells were trypsinized, washed with PBS, and fixed with 70% ethanol overnight at 4 °C. The fixed cells were washed with PBS and stained with propidium iodide (PI) for 15 min in the dark. Then, the cell cycle arrest was analyzed using an Epics XL-MCL flow cytometer (Beckman Coulter, Miami, FL, USA). The treated and untreated cells were trypsinized, washed with PBS, and costained with Annexin V and PI for 10 min, respectively. The apoptosis of cells was analyzed using an Epics XL-MCL flow cytometer (Beckman Coulter).

### 2.9 Western blot analysis

The effects of HepG2 treated with LR042 and/or radiation on the expression levels of proteins associated with different signaling pathways were examined using Western blot analysis.^29^ The total cellular proteins were extracted by incubating the cells in a lysis buffer obtained from Cell Signaling Technology. Protein concentrations were determined using BCA assay. P53, Bax and Bcl-2 caspase-3 and AKT were purchased from Abcam, Cell Signaling Technology, and Proteintech. SDS-PAGE was performed in 10% tricine gels, and equal amounts of protein were loaded per lane. The procedure was conducted as described previously. After electrophoresis, the separated proteins were transferred to nitrocellulose membranes and blocked with 5% nonfat milk in TBST buffer for 1 h. Thereafter, the membranes were incubated with primary antibodies at 1 : 1000 dilutions in 5% nonfat milk overnight at 4 °C, and then secondary antibodies were conjugated with horseradish peroxidase at 1 : 2000 dilution for 1 h at room temperature.

### 2.10 Immunofluorescence

HepG2 cells in complete growth medium at 5 × 10^4^ cells per mL were incubated with LR042 (5 μM) for 24 h, unless otherwise stated. The cells were washed once in PBS, fixed, permeabilized simultaneously using 4% paraformaldehyde with 1% Triton X-100 in PBS, quenched with 0.1 M glycine in PBS, and blocked overnight at 4 °C with 3% (w/v) BSA. The fixed cells were stained with primary antibodies as indicated.^30^ Cell morphology was observed using a laser confocal microscope.

## 3. Results and discussion

### 3.1 Distribution model of the 125I seed brachytherapy source

Either intracavitary or implantation brachytherapy needs an accurate localization of the position of radiation source to calculate the dose distribution. The dosimetric properties of brachytherapy sources can be obtained by calculating the dosimetric parameters. The optimization of dose distribution is important in brachytherapy. As shown in Scheme 1B, the ^125^I seeds were arranged uniformly on the 96-well plate. Four ^125^I seeds were positioned in one well with a fixed distance (0.9 cm) from one another. According to the literature, the radiation dose of every well on the 96-well plate was calculated for 0.57 cm. The radiation energy of every ^125^I seed was 0.992 ± 0.025 cGy h^−1^ U^−1^. After treatment with ^125^I seed for 72 h, the accumulated radiation dose of the tumor cells reach 285.7 ± 7.2 cGy U^−1^.^31^

### 3.2 Radiotherapy sensitization of LR042 to enhance ^125^I-seed-induced growth inhibition

The antiproliferative activities of LR042 and DR042 were screened using MTT assay against human hepatocellular liver carcinoma HepG2 cells, human colon cancer SW480 cells, and human lung adenocarcinoma A549 cells. The inhibitory activities (IC_50_) of the complex against different tumor cells are listed in Table 1. It is found that laevo-isomer LR042 displayed great growth inhibition against HepG2 cells (IC_50_ = 9.63 μM) and SW480 cells (IC_50_ = 1.31 μM) after 72 h of treatment. However, dextro-isomer DR042 exhibited little suppression on different tumor cells. These data suggested that LR042 displayed much more promising inhibitory effect against a variety of tumors cells than DR042 (Table S1 in ESI†). However, dextro-isomer DR042 exhibited little suppression on different tumor cells. These data suggested that LR042 displayed much more promising inhibitory effect against a variety of tumor cells than DR042.

**Table 1:** Inhibitory effect (IC_50_, μM) of LR042 and ^125^I on human cancer cells after 72 h of treatment

Comp.	IC_50_ (μM)			
	HepG2	SW480	A549	HaCaT
LR042	9.63 ± 1.49	1.13 ± 0.32	>20	9.65 ± 0.21
LR042 with ^125^I	1.81 ± 0.50	0.79 ± 0.90	>20	10.61 ± 0.38
Radiosensitivity index^a^	5.32	1.43	—	0.91

a Radiosensitivity index = IC_50_ (LR042)/IC_50_ (LR042 with ^125^I) for different cells.

**Fig. 1:**
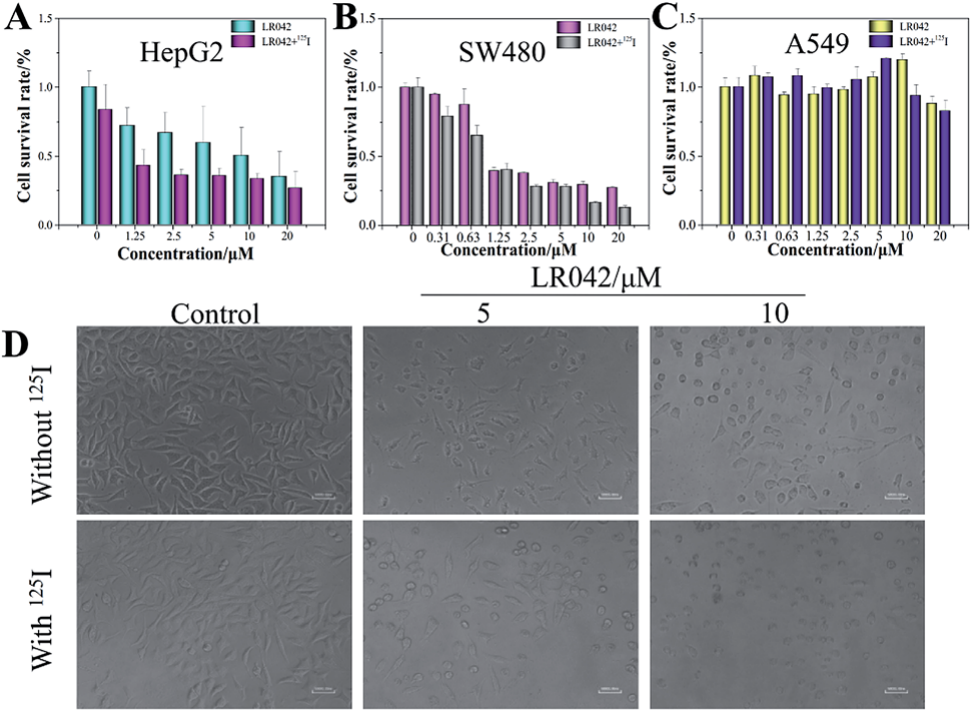
LR042 enhanced ^125^I radiation-induced HepG2 cell (A), SW480 cell (B), and A549 cell (C) growth inhibition. These tumor cells were pretreated with various concentrations of LR042 (0–20 μM) for 72 h and treated with and without ^125^I. (D) Changes in the morphology of cells exposed to LR042 with and without ^125^I radiation were captured using fluorescence microscopy (×40 magnification).

However, with continuous and low-dose radiation with ^125^I seeds for 72 h, the inhibitory activities of LR042 against HepG2 and SW480 cells decreased markedly to 1.81 and 0.79 μM, respectively. As shown in Fig. 1, for HepG2 cells, LR042 inhibited the growth of HepG2 cells with increasing concentration. Then, the inhibitory effect of ^125^I radiation effectively increased. However, for the SW480 and A549 cells, little improvement by ^125^I radiation was observed. Synthetic LR042 enhanced the radiosensitivity of HepG2 cells to ^125^I seeds. The IC_50_ values of the combined treatment against HepG2 cells dramatically decreased from 9.63 μM (^125^I seed radiation alone) to 1.81 μM. Whereas for DR042, no obvious enhanced radiosensitivity was observed against various tumor cells (Table S1 in ESI†). The radiosensitivity index was 5.32, which suggested LR042 can induce tumor cell death at a low concentration under ^125^I radiation (Table 1). On the basis of its promising *in vitro* activity, LR042 was selected as an early lead for a preliminary evaluation in further study.

Furthermore, under the phase-contrast observation of the HepG2 cells, ^125^I seed radiation alone exerted little influence on the cell viability and morphology. Nevertheless, with the treatment of LR042 at the concentration of 5 and 10 μM for 72 h, the HepG2 cells displayed loss of cell-to-cell contact, dose-dependent cell shrinkage, and reduction in cell number. Then, when LR042 was combined with ^125^I seed radiation, the cell morphology profoundly shrank to a round shape, which indicated HepG2 cell death, in a special incubation of LR042 (10 μM) and ^125^I (Fig. 1D). The evident reduction of cell viability and marked cell morphology change indicated that LR042 can effectively enhance the inhibitory activity of ^125^I seeds against the growth of tumors cells.

### 3.3 Drug distribution and localization

**Fig. 2:**
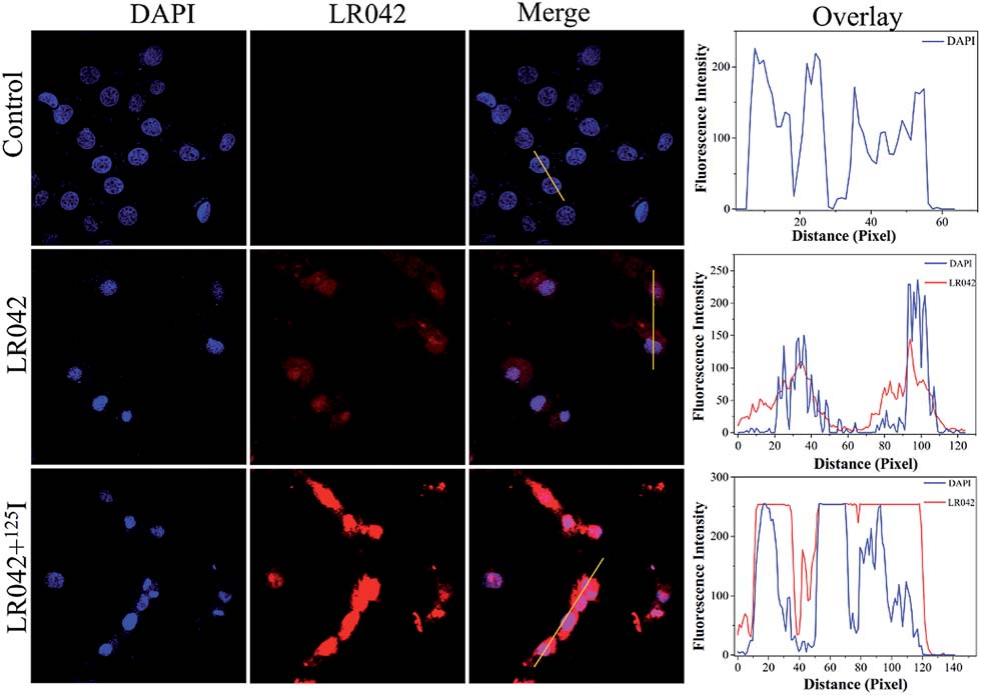
Cellular localization and distribution of LR042 in the HepG2 cells. The cells were treated with LR042 and LR042–^125^I for 24 h at 37 °C: blue, DAPI; red, ruthenium(ii) complexes, [LR042] = 5 μM. Fluorescent images were viewed using a confocal laser scanning microscope.

Moreover, the cellular localization of LR042 in HepG2 cells without and with the radiation of ^125^I were further investigated, as shown in Fig. 2. Accordingly, the ability of LR042 to membrane-permeabilized cells was characterized by employing confocal laser scanning microscopy (CLSM). As shown in Fig. 2, the nucleus of HepG2 cells was highlighted to blue by the DNA dye DAPI (4′,6-diamidino-2-phenylindole). Without the drug, the nucleus was round and plump. After treatment, LR042 emitted red fluorescence that distributed in the whole cell, mainly enriched in nucleus and a little distributed in the cytoplasm. Moreover, combined with ^125^I radiation, LR042 mainly distributed in the whole cell with strong red fluorescence, and the cell nucleus notably shrank and condensed into smaller balls. These results suggested that LR042 may cause cell death by inducing DNA damage.^32^

### 3.4 Biochemical mechanism studies

#### 3.4.1 LR042 enhanced apoptosis induced by ^125^I radiation

**Fig. 3:**
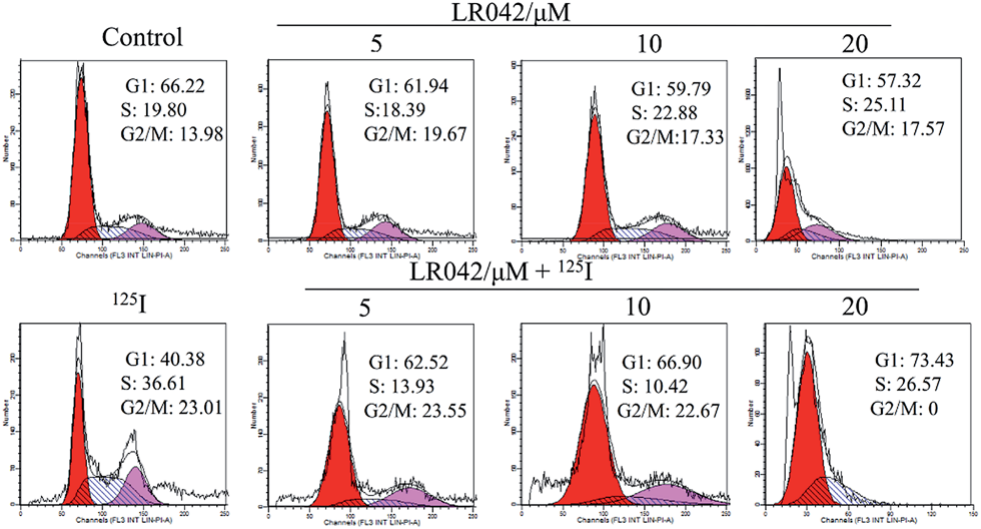
Cell cycle distribution of HepG2 cells incubated with LR042 with and without ^125^I radiation by using flow cytometric analysis. The cells were treated with LR042 (0, 5, 10 and 20 μM) and cotreated with and without ^125^I radiation for 72 h.

Furthermore, flow cytometry was performed to examine the inhibitory activity of LR042 and the combined treatment of LR042–^125^I against the growth of HepG2 cells. Specifically, the suppression of cancer cell proliferation resulted from apoptosis, cell cycle arrest, or the joint action of both modes (Fig. 3).^33^ As shown in Fig. 3, the exposure of HepG2 cells to 0, 5, and 10 μM LR042 for 24 h exerted no evident influence to the cell cycle. Under ^125^I seed radiation, the number of cells under S-phase arrest significantly increased. However, the combined LR042–^125^I treatment, did not exert evident changes to the cell cycle. These results indicated that LR042 and the combination of LR042–^125^I did not cause tumor cells death by interfering the cell cycle.^34,35^

**Fig. 4:**
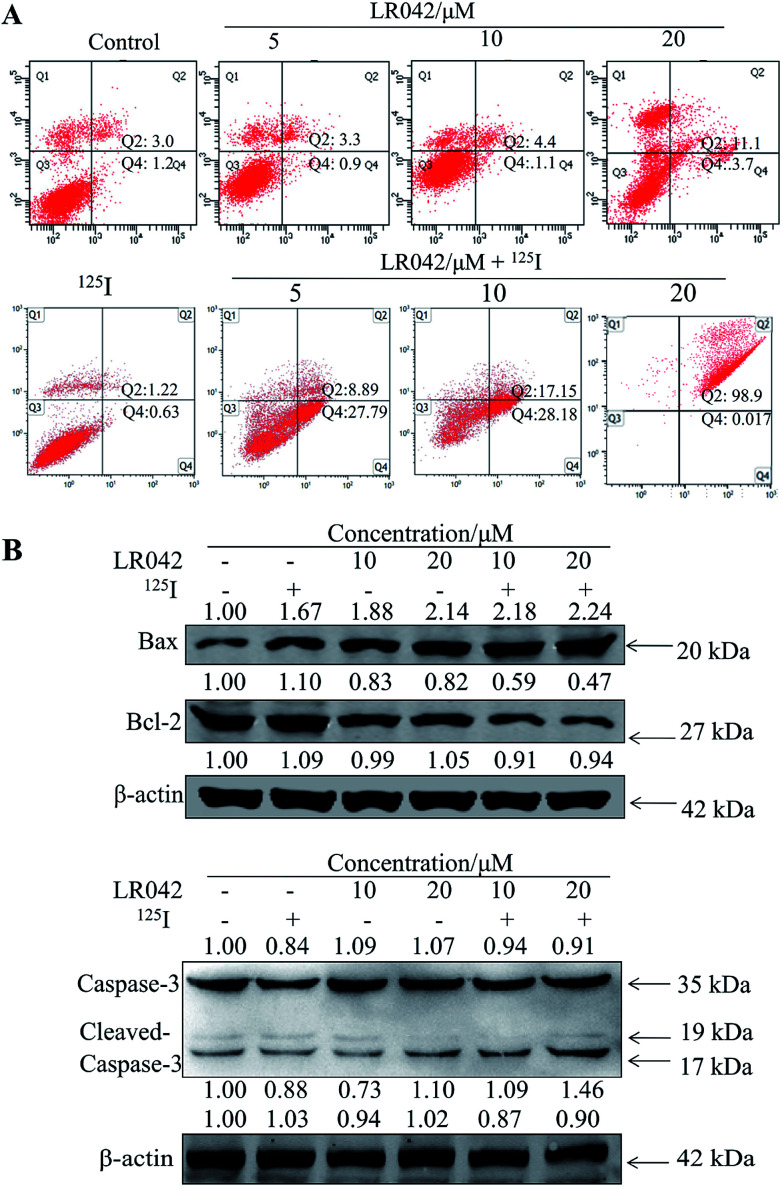
(A) LR042 enhanced ^125^I-induced HepG2 cell apoptosis. The cells were incubated with LR042 (0, 5, and 10 μM) and cotreated with and without ^125^I radiation for 72 h. (B) LR042 enhanced the ^125^I-induced apoptosis of HepG2 cells and the related signaling pathways, which were examined using Western blot analysis. The cells were treated with LR042 (0, 10, and 20 μM) and cotreated with and without ^125^I radiation for 72 h.

To further confirm the potential mechanism of the combination of LR042–^125^I seeds, flow cytometry was also performed to analyze cell apoptosis by staining the cells with Annexin V and PI. As indicated in Fig. 4, increasing concentrations of free LR042 slightly increased both the early (Q4 region) and late (Q2 region) stages of apoptosis. At 10 μM concentration of LR042, 1.1% of the cells were in the early apoptotic stages and 4.4% in the late stages. However, with the addition of ^125^I seed radiation at a concentration of 10 μM LR042, 28.18% of the cells were in early apoptosis and 17.15% in late apoptosis. LR042 may effectively enhance ^125^I-induced inhibitory activity against the growth of HepG2 cells *via* apoptosis.

To investigate the underlying mechanisms for the cotreatment-induced apoptosis, the activation of Bcl-2 and Bax were confirmed using Western blot analysis. As shown in Fig. 4B, some certain up-regulate of Bax and down-regulate of Bcl-2 with the radiation of ^125^I were observed. Moreover, with the addition of LR042 combined with ^125^I, the up-regulate of Bax and down-regulate of Bcl-2 were enhanced notably. It is well known that Bax is a key pro-apoptotic protein which the increasing expression followed with apoptosis, and Bcl-2 is an important inhibitory apoptosis protein which the decreasing expression followed with apoptosis. As a marker of apoptosis, caspase-3 can be cleaved and activated during apoptosis.^36^ As shown in Fig. 4B, cotreatment with LR042 induced the activation of caspase-3 in HepG2 cells after 72 h of treatment as evidenced by the appearance of cleaved and increased levels of caspase-3 (17 kDa) in comparison with the single treatment of ^125^I radiation. No changes were detected in the expression of total caspase-3. These results indicated that the enhancement of the HepG2 cell apoptosis contributed to the synergistic effects of LR042 and ^125^I radiation.

#### 3.4.2 LR042 enhanced ^125^I-induced DNA damage and related signaling pathways

**Fig. 5:**
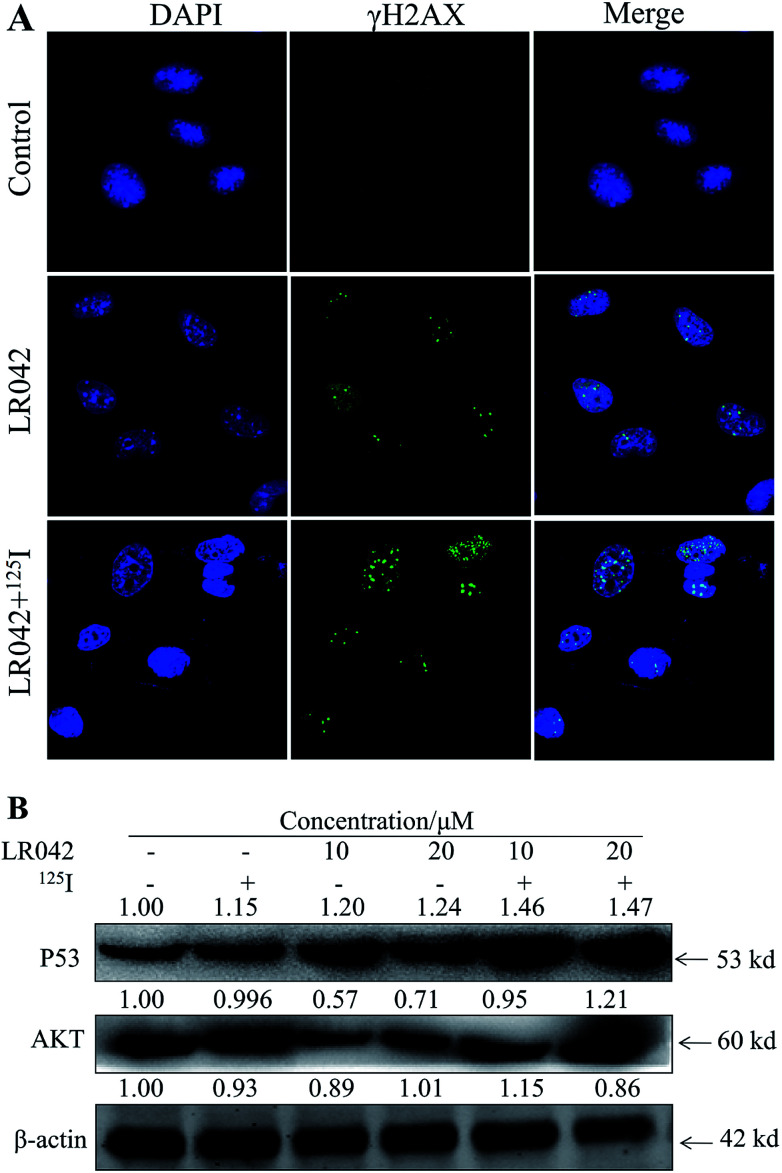
(A) LR042 enhanced the ^125^I-induced DNA damage of the HepG2 cells. The cells were treated with LR042 and LR042–^125^I for 24 h at 37 °C. The confocal immunofluorescence assays (IF) of HepG2 cells were dealed with antibodies specific to DSBs biomarker γH2AX, [LR042] = 5 μM. (B) LR042 enhanced the ^125^I-induced apoptosis of HepG2 cells and related signaling pathway. The DNA damage and related signaling pathways were examined using Western blot analysis. The cells were treated with LR042 (0, 10, and 20 μM) and cotreated with and without ^125^I radiation for 72 h.

DNA is the most important and sensitive target molecule for radiation biological effects. DNA damage as the main mechanism for the combined treatments of radiotherapy and chemotherapy is reported.^37^ In this study, the induction of DNA double-strand breaks (DSBs) in HepG2 cells were investigated using confocal immunofluorescence assays (IFs) and staining with γH2AX (an early marker of DNA damage response).^38^ HepG2 cells showed little activation of γH2AX in response to LR042 treatment. However, the combination of LR042–^125^I seeds (Fig. 5A) induced an increasing number of γH2AX-positive cells. The enhancement of DNA damage contributed to the synergistic effects of LR042 and ^125^I radiation in HepG2 cells.

Moreover, P53, a classic DNA damage response marker, is activated by AKT phosphorylation.^39^ AKT, also called protein kinase B, is described as the critical upstream mediator of wild-type p53 and is known to suppress DNA replication. The proapoptotic ability of p53 is activated through the expression of AKT. Given the role of the AKT–p53 axis in triggering DNA-damage-induced apoptosis, this study examined the protein levels of AKT and p53 in response to treatment with free LR042 and the combination of LR042–^125^I seeds. Cellular extracts from the HepG2 cells revealed an increase in AKT–p53 protein.^40^

**Fig. 6:**
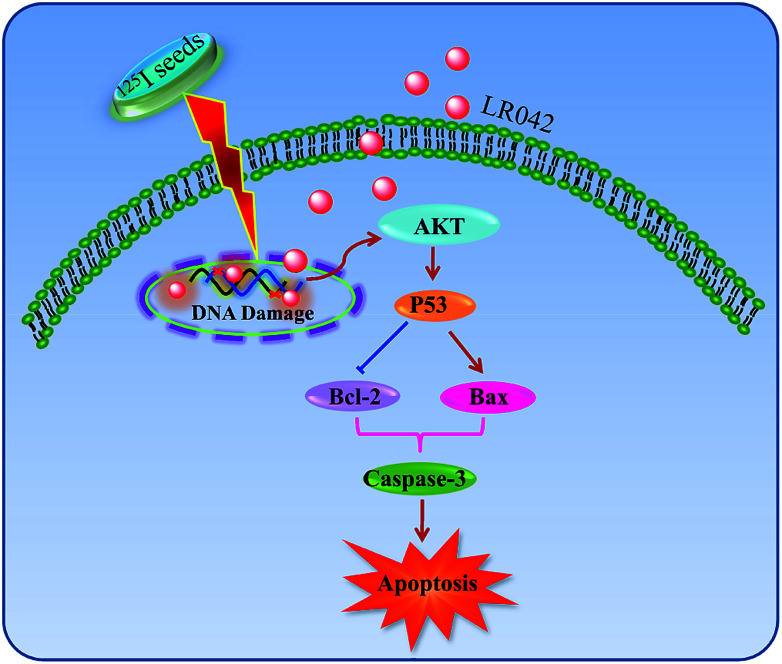
Proposed signaling pathways of apoptosis induced by LR042–^125^I cotreatment. LR042 enhanced the radiosensitivity of cells to ^125^I-induced DNA damage. The activation of the p53 pathway further activated AKT and caspase-3 and resulted in the apoptosis of HepG2 cells.

As shown in Fig. 5B, after treatment with free LR042, the expression level of p53 increased evidently, whereas little significant change was observed with free ^125^I radiation. Furthermore, the up-regulation of p53 was notably observed after the combined treatments (LR042–^125^I radiation). LR042 can enhance the I^125^-induced DNA damage, up-regulate the expression of p53, and further induce the apoptosis of HepG2 cells. The protein serine/threonine kinase AKT plays essential roles on the regulation of cell proliferation and survival.^41^ Activated p53 up-regulation of AKT may lead to an irreversible commitment to apoptotic cell death.^42^ As shown in Fig. 5B, alone LR042 can decrease the expression level of AKT, but the combination of LR042 with ^125^I seed radiation can markedly increase the expression of AKT. LR042 can serve as a potential radiosensitizer to induce apoptosis of HepG2 cells through the DNA-damage-activated AKT–p53 pathway (Fig. 6).

## 4. Conclusions

In this study, it is found that laevo-isomer of chiral ruthenium(ii) polypyridyl complex (LR042) exhibited much more better inhibition than dextro-isomer (DR042). Moreover, LR042 could enhance the I^125^-induced suppression on HepG2 cells through the induction of apoptosis by accelerating DNA damage. Further study indicated the occurrence of DNA damage and activated downstream signaling pathways, including up-regulation of the p53 and AKT, thereby finally resulting in an increase of radiation sensitivity and inhibition of tumor cells proliferation. The synthetic LR042 can be further developed as radiosensitizer of ^125^I by inducing DNA-damage-mediated apoptosis for cancer therapy.

## Conflicts of interest

The authors declare no competing financial interests.

## Supplementary Material

RA-008-C8RA03383H-s001
