# Supplementary material for: Chiral ruthenium(ii) complex as potent radiosensitizer of 125I through DNA-damage-mediated apoptosis
Source: RSC Adv. 2018 Jun 6;8(37):20612–8. doi: 10.1039/c8ra03383h (PMC9080800; doi:10.1039/c8ra03383h)
Supplement: RA-008-C8RA03383H-s001 [file RA-008-C8RA03383H-s001.pdf]

Electronic Supplementary Material (ESI) for RSC Advances.
This journal is © The Royal Society of Chemistry 2018

Supporting Information

for

Chiral Ruthenium(II) Complex as Potent Radiosensitizer of ^{125}I through DNA-Damage-Mediated Apoptosis

Mingjun Bai[a#], Shaolin Zeng[a#], Li Li[b], Qiong Wu[b*], Yanyang Zhang[a], Tao Pan[a], Luwen Mu[a], Duo Zhu[a], Shouhai Guan[a*], Qiang Xie[a*], Wenjie Mei[b]

[a]*Department of Vascular Interventional Radiology, the Third Affiliated Hospital, Sun Yat-sen University,600 Tianhe Road, Guangzhou, Guangdong, China；510630*

[b] *School of Pharmacy, Guangdong Pharmaceutical University, Guangzhou, China：510006*

Corresponding Author:E-mail: xieqiangdoctorxie@126.com; Guanshouhai1148598268@qq.com; wuqiongniu.1113@163.com

[#] *These authors contributed equally to the work*

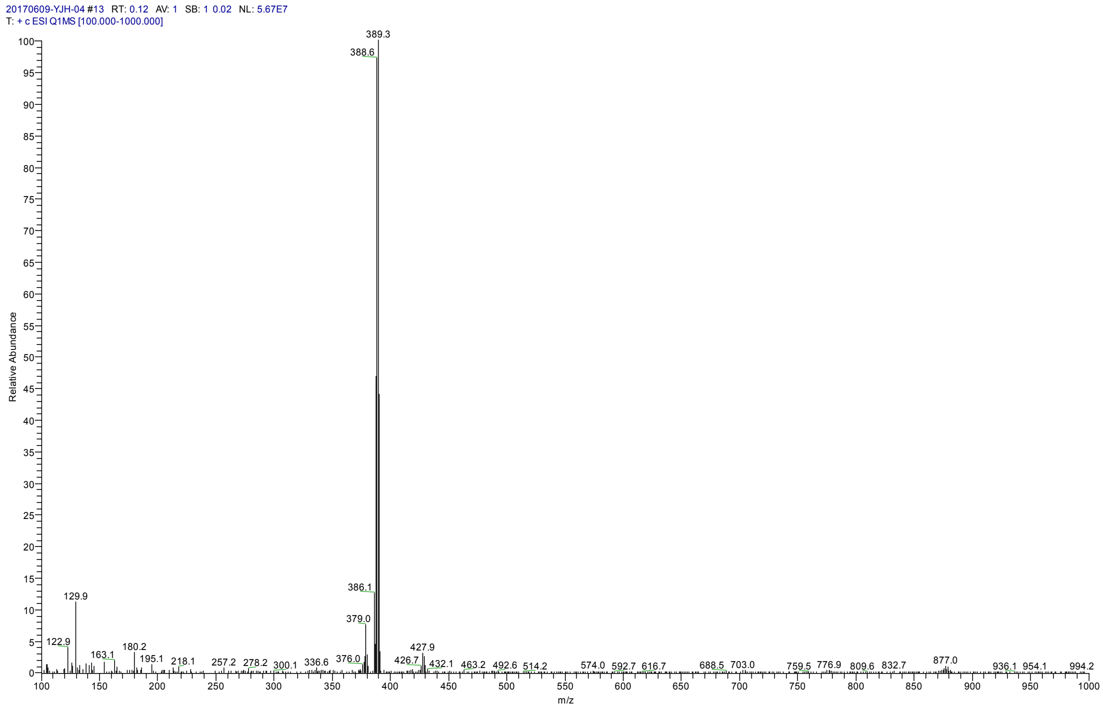

A

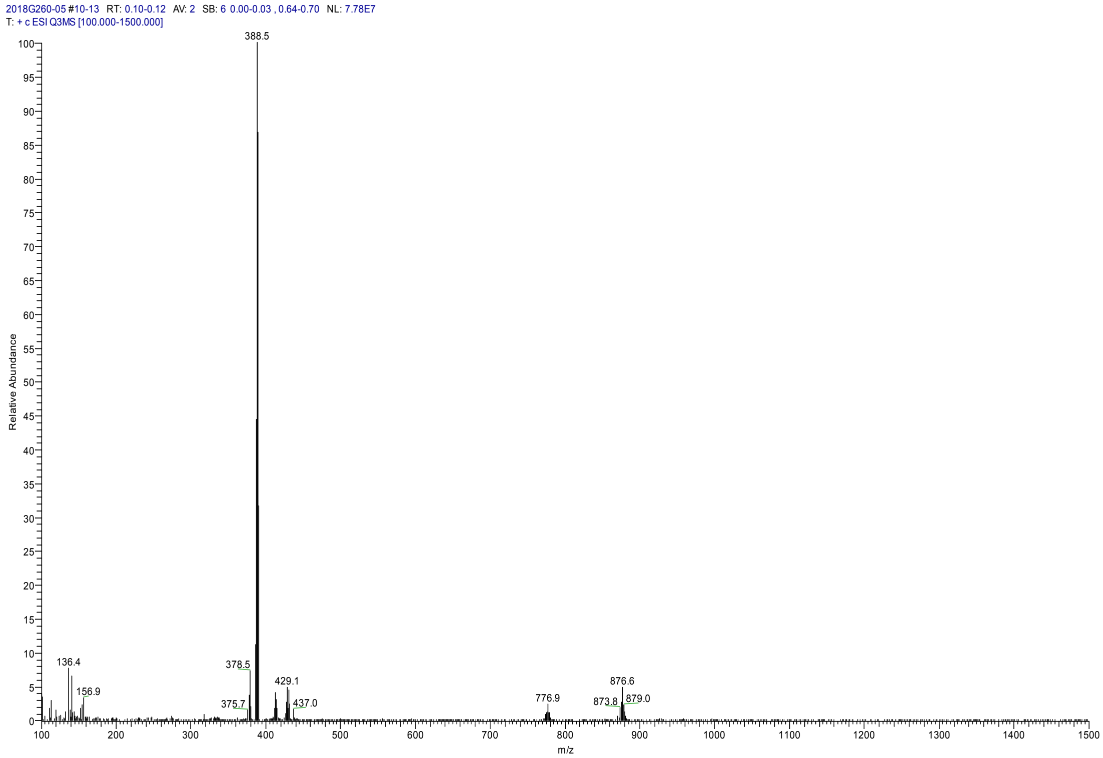

B

Figure S1. The ESI-MS spectra of LR042 (A) and DR042 (B)

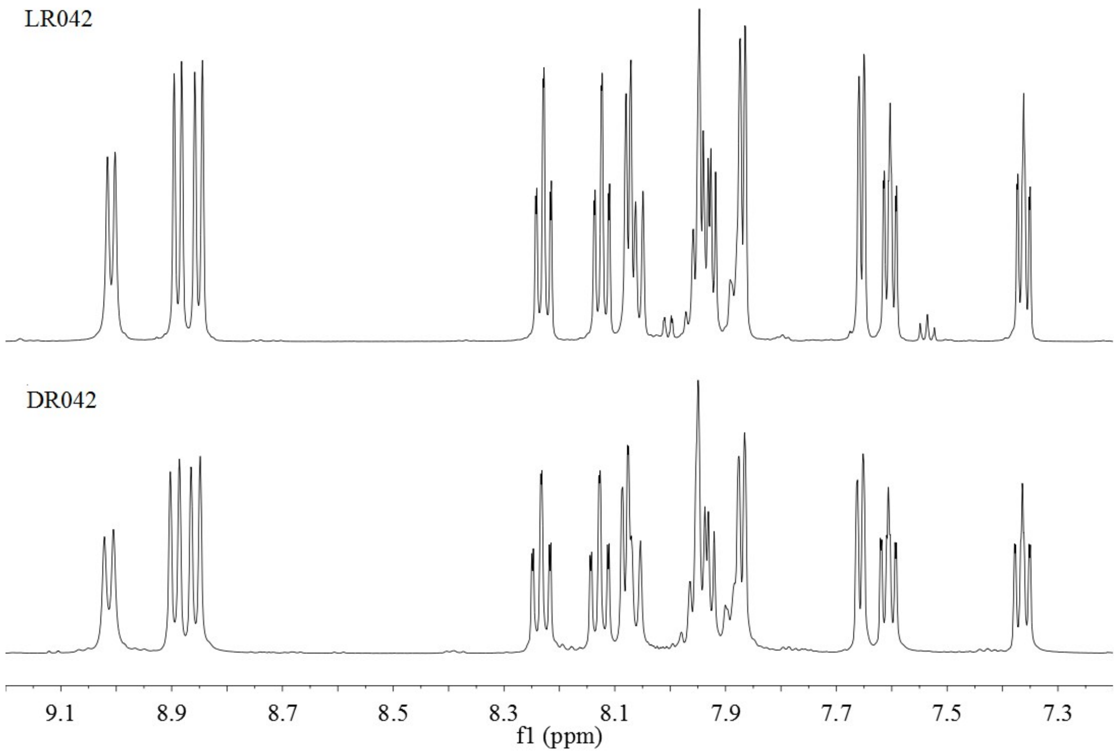

Figure S2. The ^{1}H NMR spectra of LR042 (upper) and DR042 (lower)

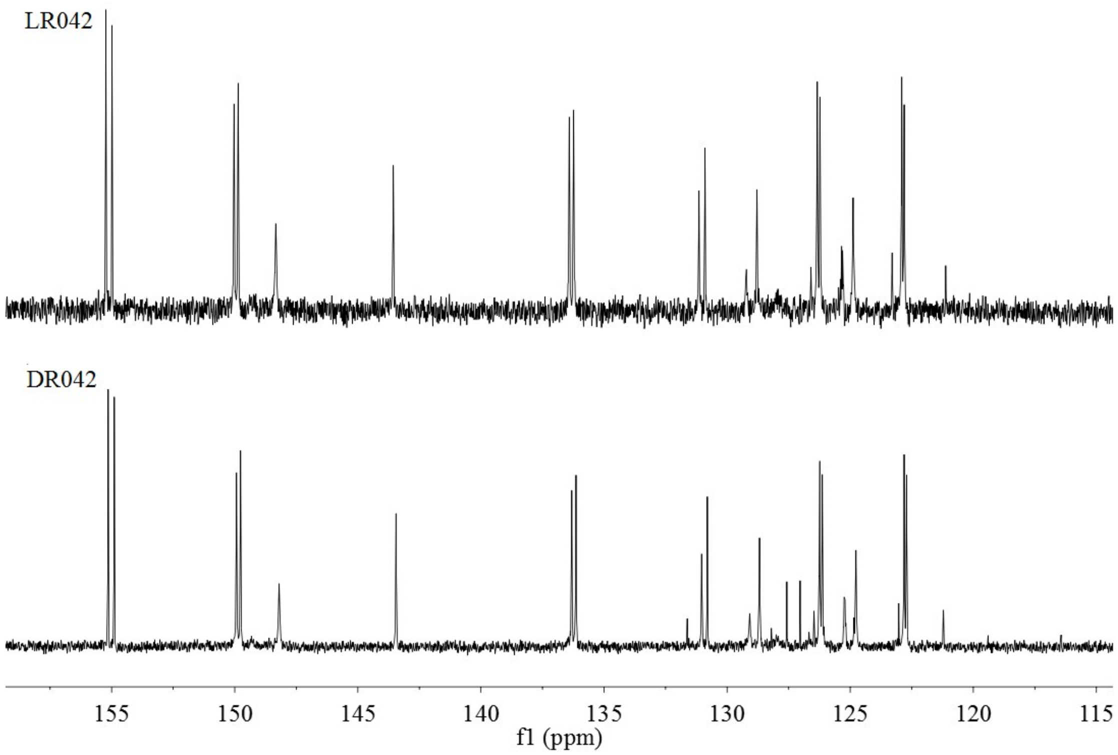

Figure S3. The ^{13}C NMR spectra of LR042 (upper) and DR042 (lower)

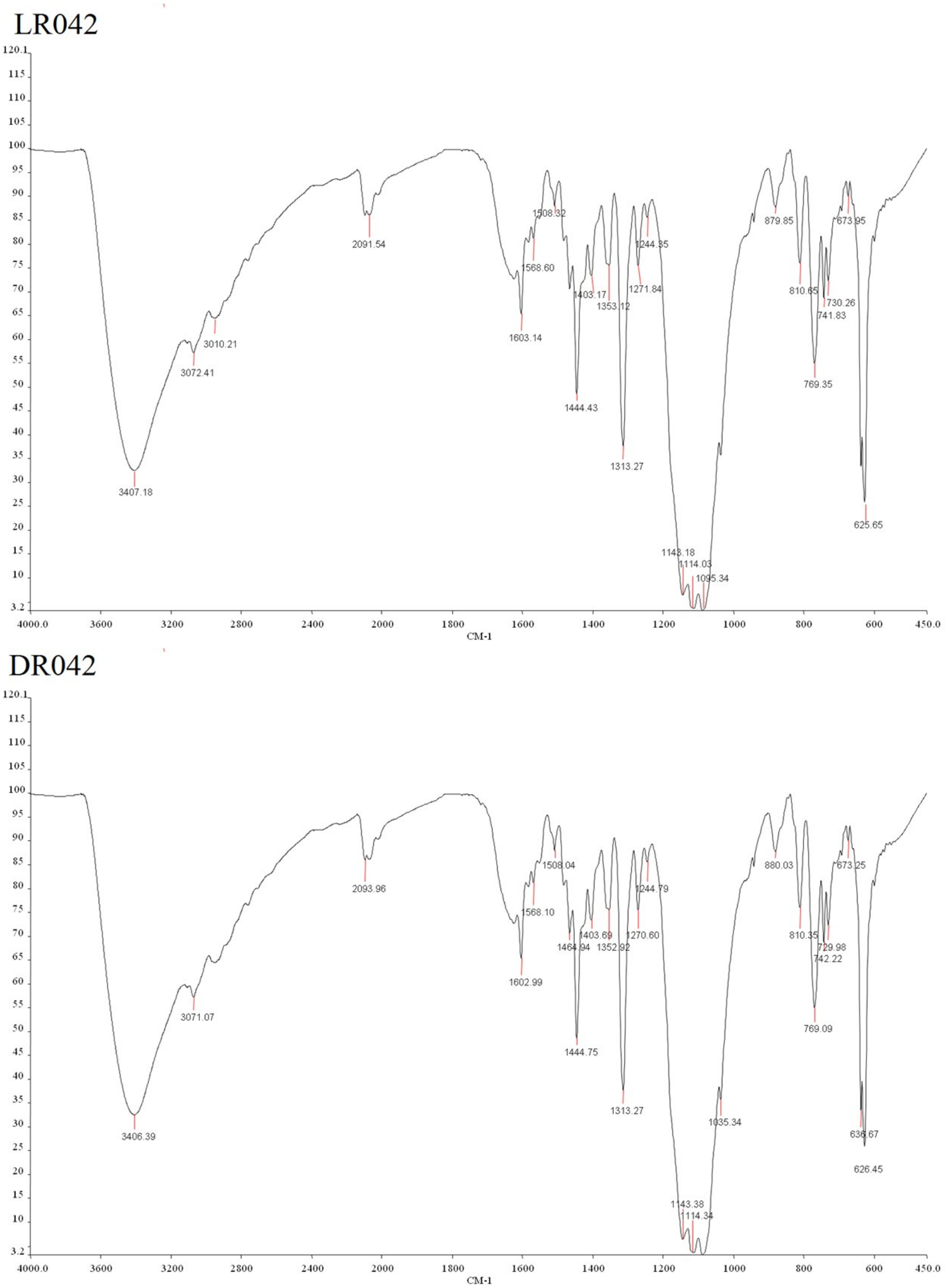

Figure S5. The IR spectra of LR042 (upper) and DR042 (lower)

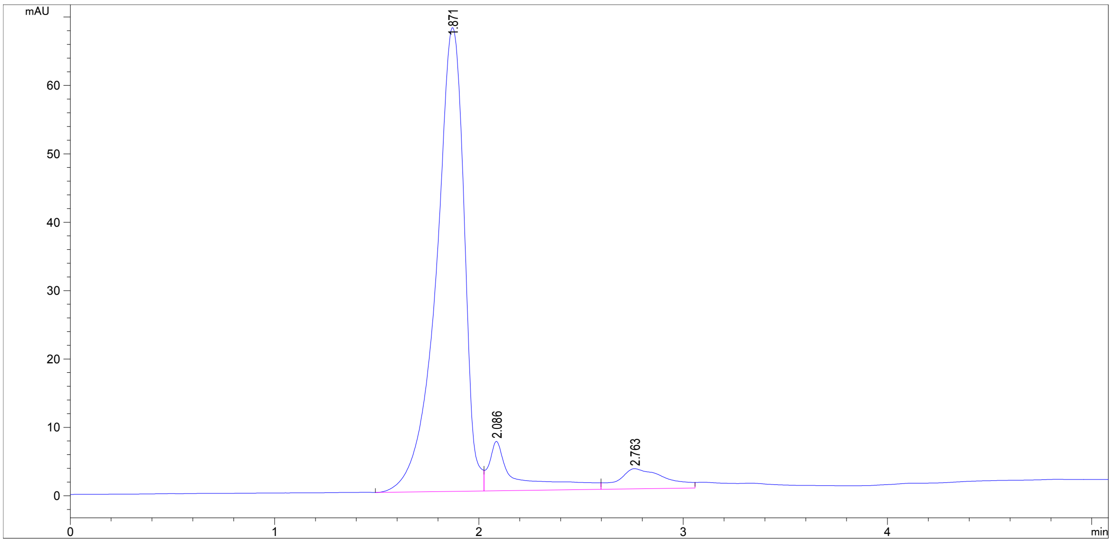

A

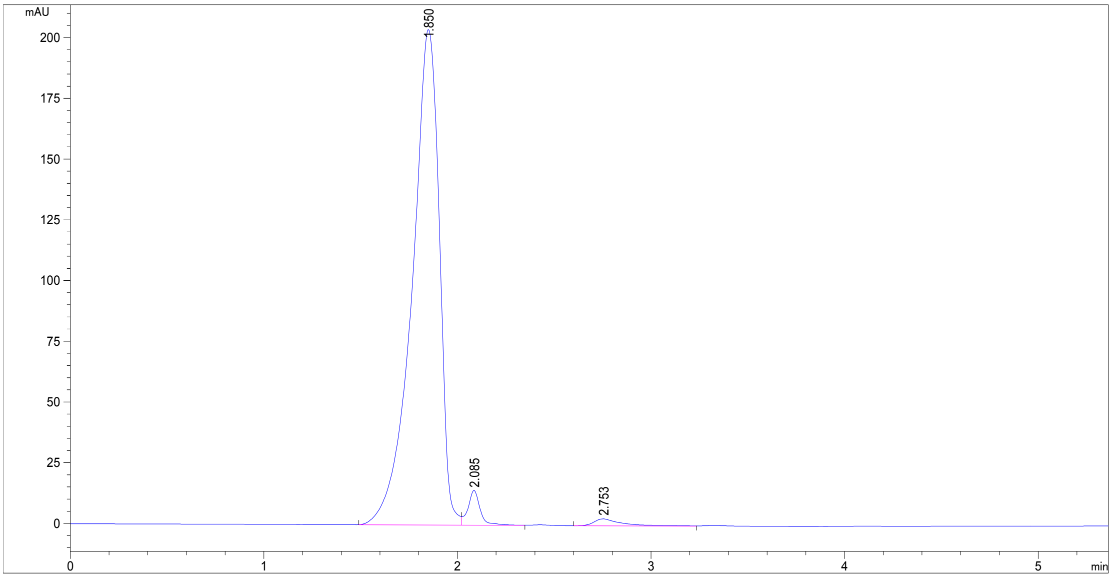

B

Figure S6. The HPLC purity analysis for LR042 (A) and DR042 (B). The eluent is MeOH:CH_3CN=1:1 for 10 min.

Table S1. Inhibitory effect (IC_{50}, μM) of DR042 and ^{125}I on human cancer cells after 72 h of treatment.

**Comp.**	IC_{50} (μM)			
	HepG2	SW480	A549	HaCaT
**DR042**	>20	>20	>20	>20
**DR042 with ^{125}I**	>20	>20	>20	>20

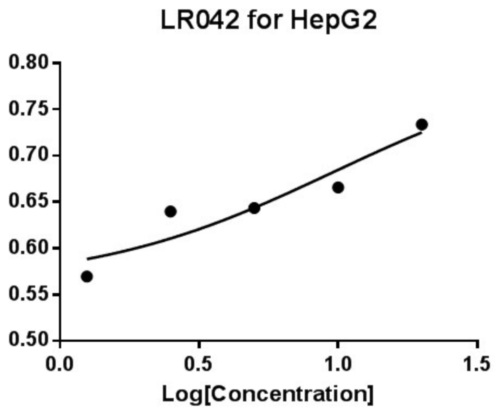

Nonlin fit	A Inhibitory rate Y
1 log(inhibitor) vs. response (three parameters)	
2 Best-fit values	
3 Bottom	0.8044
4 Top	0.5601
5 LogIC50	0.9835
6 IC50	9.627
7 Span	-0.2443
8 Std. Error	
9 Bottom	0.1370
10 Top	0.05307
11 LogIC50	0.7149
12 Span	0.1056
13 95% Confidence Intervals	
14 Bottom	0.2148 to 1.394
15 Top	0.3318 to 0.7885
16 LogIC50	-2.093 to 4.060
17 IC50	0.008078 to 11474
18 Span	-0.6985 to 0.2100
19 Goodness of Fit	
20 Degrees of Freedom	2
21 R square	0.8820
22 Absolute Sum of Squares	0.001637
23 Sy.x	0.02861

A B

Figure S7. The fitting curve (A) and calculating results (B) of LR042 treated with HepG2 cells

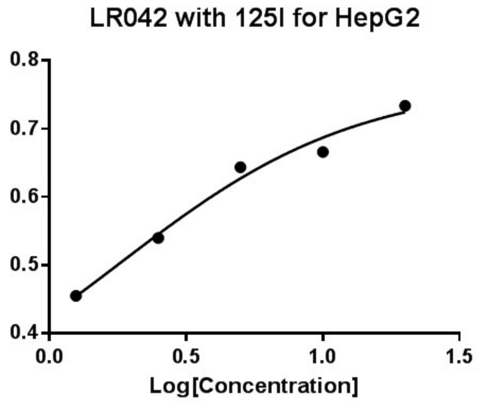

Nonlin fit	A Data Set-A Y
1 log(inhibitor) vs. response (three parameters)	
2 Best-fit values	
3 Bottom	0.7681
4 Top	0.2380
5 LogIC50	0.2583
6 IC50	1.813
7 Span	-0.5301
8 Std. Error	
9 Bottom	0.03258
10 Top	0.1449
11 LogIC50	0.2848
12 Span	0.1234
13 95% Confidence Intervals	
14 Bottom	0.6279 to 0.9083
15 Top	-0.3853 to 0.8614
16 LogIC50	-0.9672 to 1.484
17 IC50	0.1078 to 30.46
18 Span	-1.061 to 0.0008156
19 Goodness of Fit	
20 Degrees of Freedom	2
21 R square	0.9828
22 Absolute Sum of Squares	0.0008343
23 Sy.x	0.02042

A B

Figure S8. The fitting curve (A) and calculating results (B) of LR042 with ^{125}I treated with HepG2 cells

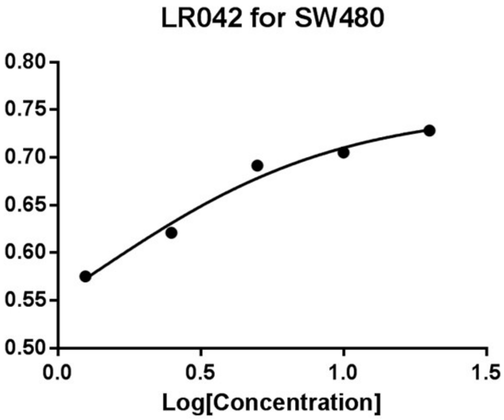

	Nonlin fit	A Inhibitory rate Y
1	log(inhibitor) vs. response (three parameters)	
2	Best-fit values	
3	Bottom	0.7501
4	Top	0.4028
5	LogIC50	0.1163
6	IC50	1.307
7	Span	-0.3473
8	Std. Error	
9	Bottom	0.01778
10	Top	0.1325
11	LogIC50	0.3377
12	Span	0.1202
13	95% Confidence Intervals	
14	Bottom	0.6736 to 0.8266
15	Top	-0.1673 to 0.9729
16	LogIC50	-1.337 to 1.569
17	IC50	0.04605 to 37.10
18	Span	-0.8644 to 0.1697
19	Goodness of Fit	
20	Degrees of Freedom	2
21	R square	0.9811
22	Absolute Sum of Squares	0.0003073
23	Sy.x	0.01240
24		

A B

Figure S9. The fitting curve (A) and calculating results (B) of LR042 treated with SW480 cells

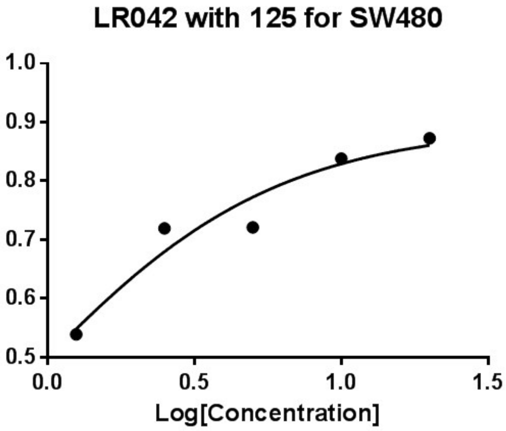

	Nonlin fit	A Inhibitory rate Y
1	log(inhibitor) vs. response (three parameters)	
2	Best-fit values	
3	Bottom	0.8947
4	Top	0.0001982
5	LogIC50	-0.1019
6	IC50	0.7909
7	Span	-0.8945
8	Std. Error	
9	Bottom	0.06079
10	Top	1.048
11	LogIC50	0.8393
12	Span	1.004
13	95% Confidence Intervals	
14	Bottom	0.6331 to 1.156
15	Top	-4.510 to 4.510
16	LogIC50	-3.713 to 3.510
17	IC50	0.0001935 to 3232
18	Span	-5.215 to 3.426
19	Goodness of Fit	
20	Degrees of Freedom	2
21	R square	0.9337
22	Absolute Sum of Squares	0.004543
23	Sy.x	0.04766

A B

Figure S10. The fitting curve (A) and calculating results (B) of LR042 with ^{125}I treated with SW480 cells

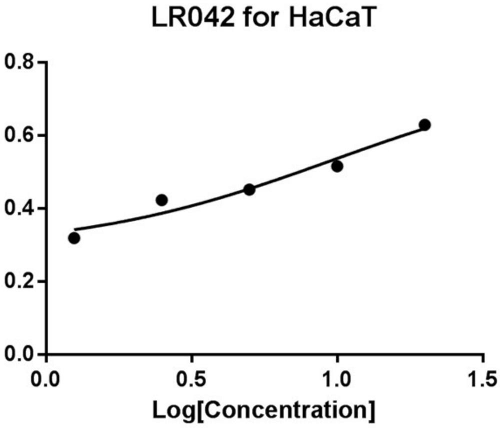

	Nonlin fit	A Inhibitory rate Y
1	log(inhibitor) vs. response (three parameters)	
2	Best-fit values	
3	Bottom	0.7792
4	Top	0.2844
5	LogIC50	0.9847
6	IC50	9.653
7	Span	-0.4948
8	Std. Error	
9	Bottom	0.1645
10	Top	0.06346
11	LogIC50	0.4230
12	Span	0.1268
13	95% Confidence Intervals	
14	Bottom	0.07136 to 1.487
15	Top	0.01135 to 0.5575
16	LogIC50	-0.8354 to 2.805
17	IC50	0.1461 to 637.8
18	Span	-1.041 to 0.05092
19	Goodness of Fit	
20	Degrees of Freedom	2
21	R square	0.9553
22	Absolute Sum of Squares	0.002347
23	Sy.x	0.03426

A B

Figure S11. The fitting curve (A) and calculating results (B) of LR042 treated with HaCaT cells.

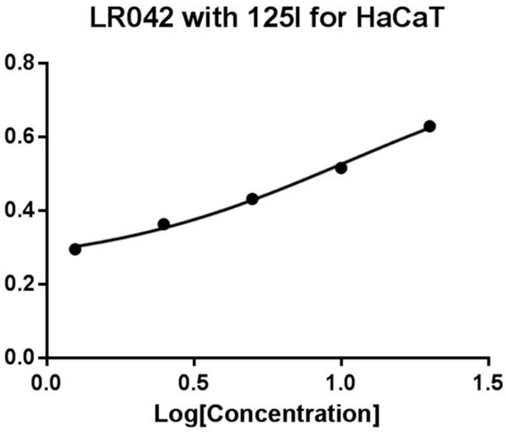

	Nonlin fit	A Inhibitory rate Y
1	log(inhibitor) vs. response (three parameters)	
2	Best-fit values	
3	Bottom	0.8279
4	Top	0.2396
5	LogIC50	1.026
6	IC50	10.61
7	Span	-0.5883
8	Std. Error	
9	Bottom	0.06387
10	Top	0.02140
11	LogIC50	0.1307
12	Span	0.05070
13	95% Confidence Intervals	
14	Bottom	0.5531 to 1.103
15	Top	0.1476 to 0.3317
16	LogIC50	0.4633 to 1.588
17	IC50	2.906 to 38.75
18	Span	-0.8064 to -0.3701
19	Goodness of Fit	
20	Degrees of Freedom	2
21	R square	0.9957
22	Absolute Sum of Squares	0.0002911
23	Sy.x	0.01206

A B

Figure S12. The fitting curve (A) and calculating results (B) of LR042 with ^{125}I treated with HaCaT cells
